# Reduced transcription of *TCOF1 *in adult cells of Treacher Collins syndrome patients

**DOI:** 10.1186/1471-2350-10-136

**Published:** 2009-12-14

**Authors:** Cibele Masotti, Camila C Ornelas, Alessandra Splendore-Gordonos, Ricardo Moura, Têmis M Félix, Nivaldo Alonso, Anamaria A Camargo, Maria Rita Passos-Bueno

**Affiliations:** 1 Centro de Estudos do Genoma Humano, Instituto de Biociências, Universidade de São Paulo, São Paulo, SP, Brazil; 2 Department of Genetics, Stanford University, California, USA; 3 Ludwig Institute for Cancer Research, São Paulo Branch, Hospital Alemão Oswaldo Cruz, São Paulo, SP, Brazil; 4 Departamento de Genética Médica, Hospital de Clínicas de Porto Alegre, Universidade Federal do Rio Grande do Sul, Porto Alegre, RS, Brazil; 5 Departamento de Cirurgia Plástica, Hospital das Clínicas da Faculdade de Medicina, Universidade de São Paulo, SP, Brazil

## Abstract

**Background:**

Treacher Collins syndrome (TCS) is an autosomal dominant craniofacial disorder caused by frameshift deletions or duplications in the *TCOF1 *gene. These mutations cause premature termination codons, which are predicted to lead to mRNA degradation by nonsense mediated mRNA decay (NMD). Haploinsufficiency of the gene product (treacle) during embryonic development is the proposed molecular mechanism underlying TCS. However, it is still unknown if *TCOF1 *expression levels are decreased in post-embryonic human cells.

**Methods:**

We have estimated *TCOF1 *transcript levels through real time PCR in mRNA obtained from leucocytes and mesenchymal cells of TCS patients (n = 23) and controls (n = 18). Mutational screening and analysis of NMD were performed by direct sequencing of gDNA and cDNA, respectively.

**Results:**

All the 23 patients had typical clinical features of the syndrome and pathogenic mutations were detected in 19 of them. We demonstrated that the expression level of *TCOF1 *is 18-31% lower in patients than in controls (*p < 0.05*), even if we exclude the patients in whom we did not detect the pathogenic mutation. We also observed that the mutant allele is usually less abundant than the wild type one in mesenchymal cells.

**Conclusions:**

This is the first study to report decreased expression levels of *TCOF1 *in TCS adult human cells, but it is still unknown if this finding is associated to any phenotype in adulthood. In addition, as we demonstrated that alleles harboring the pathogenic mutations have lower expression, we herein corroborate the current hypothesis of NMD of the mutant transcript as the explanation for diminished levels of *TCOF1 *expression. Further, considering that *TCOF1 *deficiency in adult cells could be associated to pathologic clinical findings, it will be important to verify if TCS patients have an impairment in adult stem cell properties, as this can reduce the efficiency of plastic surgery results during rehabilitation of these patients.

## Background

Treacher Collins syndrome (TCS; OMIM 154500) is a rare autosomal dominant craniofacial disorder (1:50.000) characterized by bilateral and symmetrical malformations, which frequently includes hypoplasia of the mandible and zygomatic complex, down-slanting palpebral fissures, coloboma of the lower eyelid and absence of eyelashes medial to this defect, external and middle ear malformation, and conductive hearing loss [1]. The penetrance is considered to be complete, but there is a high inter and intra-familial phenotypic variation, ranging from cases with perinatal death due to airway obstruction by severe orofacial malformations to those that are not clinically diagnosed [2].

In most of the cases the disorder is caused by frameshift deletions or duplications of 1-41 bp in *TCOF1 *coding region which cause premature termination codons (PTC). Except for a recurring 5 bp deletion in exon 24 that is responsible for 17% of the cases, mutations are usually family-specific [3]. *TCOF1 *gene product is a nucleolar protein (treacle) involved in rRNA transcription and in pre-rRNA post-transcriptional modification [4]. Truncated proteins are not detected in TCS patients' fibroblasts and lymphocytes, suggesting that mRNA bearing PTCs are being degraded by nonsense mediated mRNA decay (NMD) [5]; however, NMD has never been demonstrated in human or mouse cells with null mutations in *TCOF1*.

No genotype-phenotype correlation has been observed in TCS and there is also no evidence of association between the disease severity and parental origin or type of the pathogenic mutation, male or female sex, sporadic or familial cases [5-9].

*TCOF1 *is expressed in various adult and embryonic tissues and haploinsufficiency of treacle during embryonic development has been proposed as the molecular mechanism underlying TCS [10]. *In situ *hybridization studies of the *Tcof1 *orthologue demonstrated that there is a peak of expression of the gene in E8.5-9.5 mice embryos, especially in the first and second pharyngeal arches [11]. Critical dosage of *Tcof1 *for appropriate craniofacial development has been further demonstrated in *Tcof1+/- *mice [12,13]. Interestingly, a previous study observed that cellular amount of treacle in fibroblasts and lymphocytes derived from TCS patients was indistinguishable from that of control individuals, and the authors suggested that a dosage compensation mechanism could occur in adulthood to compensate the null allele [5]. However, until the present, these findings have not been confirmed.

The relevance of *TCOF1 *happloinsufficiency during adult life is still rarely studied: it can predispose to age related macular degeneration [14] and explain the absence of long-term stable results in mandibular distraction for facial reconstruction [15-17]. These findings might imply that *TCOF1 *adult stem cells present a decreased regenerative capacity in comparison to wild-type cells. We have therefore conducted the present study to verify if *TCOF1 *expression is altered in adult TCS cells as well as to identify a source of human tissue suitable for functional studies of treacle in adulthood.

## Methods

### Patients and controls

We studied peripheral blood samples from 20 TCS patients (TCS1 to 20, ranging from 3 to 41 years old) referred to our center for genetic counseling and 12 controls (ranging from 20 to 50 years old). We also obtained tissue samples from TCS16, from three other patients (TCS21 to 23), and from six controls submitted to reconstructive plastic surgery at University of São Paulo Medical School. The study was approved by the ethical committee of our Institution and informed consent was obtained from both patients and control subjects or from their legal tutors. In total, 23 TCS patients were studied.

### Isolation of cells from periosteum of TCS patients

During corrective surgery, overlying periosteum from the face of four TCS patients (two males and two females aged from 6 to 29 years) was meticulously dissected away from surrounding tissues to isolate intact periosteal flaps. Control periosteum was obtained using the same procedure from facial region of six subjects (four males and two females aged from 11 months to 20 years) with no evidence of bone disease during corrective surgery.

Mesenchymal cells were isolated as described by previous reports from our group [18,19]. The periosteal flaps were thoroughly washed with sterile phosphate-buffered saline (PBS) supplemented with 4% antibiotics (100 units/mL penicillin and 100 mg/mL streptomycin; Invitrogen), and digested with trypsin solution (TrypLe; Invitrogen) for 1 h at 37°C. Once digested, the tissue was transferred with minimal dissection into 35 mm Petri dishes (Corning, NY) containing Dulbecco's modified Eagle's medium (DMEM; GIBCO) with 10% fetal bovine serum (FBS; GIBCO), 100 units/mL penicillin, and 100 mg/mL streptomycin (Invitrogen). After two weeks, cells were washed with PBS, then dissociated in trypsin solution and seeded at 10^4 ^cells per 25 cm^2 ^for the first passage. For expansion, cells were cultured in monolayer in growth medium at 37°C in a humidified atmosphere of 5% CO2. The medium was replaced every 3 days. In order to prevent cell differentiation, cultures were maintained semiconfiuent and subcultured every 4-5 days with daily medium changes.

### Flow cytometry

Cells were harvested with TrypLe (Invitrogen), washed with PBS, and incubated at 4°C for 30 min with the following anti-human antibodies: CD29-PE CY5, CD90 (Thy-1), CD45-FITC, CD73, CD105, CD117, CD 31-PE (Becton Dickinson) and SH3 (Case Western Reserve University). After the wash, unconjugated primary antibodies were incubated with anti-mouse-PE secondary antibody (Guava Technologies) for additional 15 min at 4°C. Finally, the cell suspension was washed with PBS, and 10^4 ^labeled cells were acquired with an EasyCyte flow cytometer (Guava Technologies). Control samples were incubated with PBS instead of primary antibody, followed by incubation with anti-mouse-PE secondary antibody. All the generated plots were analyzed in Guava ExpressPlus software (Guava Technologies).

### DNA and RNA isolation and cDNA synthesis

Genomic DNA from peripheral blood samples was obtained according to reference [20] and genomic DNA from culture cells was extracted using NucleoSpin Tissue extraction kits (Macherey-Nagel). Total RNA was isolated from leucocytes and mesenchymal cells using TRIzol^® ^(*Gibco BRL*), treated with *DNAse *(*Promega*) and submitted to reverse transcription using *Superscript II Reverse Transcriptase *(*Gibco BRL*), according to manufacturer's protocol. Aliquots from *DNAse *treated RNAs were used for amplification of an intronic region of *MLH1 *gene as a control for DNA contamination (primers sequences on request).

### Mutation screening

Seven of the 23 patients included in the present report were previously studied by our group [3,9,21]. Pathogenic mutations were identified in six of these seven individuals (Table 1). Except for patient TCS17, for whom we did not have enough DNA, all the other 15 patients were submitted to molecular analysis to identify the pathogenic mutation. Screening of *TCOF1 *mutations by direct sequencing of genomic DNA was performed with primers described elsewhere [9]. Sequencing of the patients' cDNA was performed in identical conditions, but using primers that amplify only the coding region flanking the pathogenic mutation (see Additional file 1).

**Table 1:** Pathogenic mutations of TCS patients analyzed for *TCOF1 *expression:

DNA and RNA from leucocytes					
**Patient**	**Pathogenic Mutation** **cDNA major isoform** **reference (AY460334)**	**genomic** **reference (NT_029289.10)**	**protein major isoform** **reference (AAR87774)**	**Screened in the present study?**	**Reference**

TCS1	(intron 6) c.639+1G>A	g.11932G>A	disrupted splicing	**Yes**	New

TCS2	(exon 23) c.4061delC	g.38890delC	p.Pro1354fs	No	[21]

TCS3	(exon 10) c.1609C>T	g.17788C>T	p.Gln537X	**Yes**	New

TCS4	(exon 14)c.2478G>A	g.21371G>A	disrupted splicing	No	[6,9]

TCS5	(exon 23) c.3853delC	g.38682delC	p.Gln1285fs	**Yes**	New

TCS6	(exon 18) c.3053_3054delGA	g.32222_32223delGA	p.Arg1018fs	No	[9]

TCS7	(exon 18) c.3053_3054delGA	g.32222_32223delGA	p.Arg1018fs	No	[9]

TCS8	(exon 24) c.4366_4370delGAAAA	g.40706_40710delGAAAA	p.Lys1457fs	No	[7,9,21]

TCS9	Not detected	-	-	No	[3]

TCS10	(exon 8) c.1095_1096delAG	g.16957_16958delAG	p.Gly366fs	**Yes**	[24]

TCS11	(exon 8) c.1095_1096delAG	g.16957_16958delAG	p.Gly366fs	**Yes**	[24]

TCS12	(exon 24) c.4361_4365delAAAAA	g.40701_40705delAAAAA	p.Lys1454fs	No	[7,21]

TCS13	(exon 9) c.1298delC	g.17302delC	p.Ala433fs	**Yes**	New

TCS14	Not detected	-	-	**Yes**	-

TCS15	Not detected	-	-	**Yes**	-

TCS16	(exon 3) c.218_222insAACC	g.6495_6499insAACC	p.Ala73fs	**Yes**	New

TCS17	Not screened	-	-	-	-

TCS18	(exon 24) c.4375_4377delAAG	g.40715_40715delAAG	p.Lys1459del	**Yes**	New

TCS19	(exon 12) c.2103_2106delTGAG	g.18620_18623delTGAG	p.Ser701fs	**Yes**	[21]

TCS20	(exon 12) c.2103_2106delTGAG	g.18620_18623delTGAG	p.Ser701fs	**Yes**	[21]

**DNA and RNA from mesenchymal cells**					

TCS16	(exon 3) c.218_222insAACC	g.6495_6499insAACC	p.Ala73fs	-	-

TCS21	(exon 24) c.4344dupA	g.40684dupA	p.Arg1448fs	**Yes**	New

TCS22	(exon 5) c.431delC	g.11097delC	p.Thr144fs	**Yes**	New

TCS23	(exon 23) c.4218dupG	g.39047dupG	p.Ser1407fs	**Yes**	[9]

### Real Time PCR

Real time quantitative PCR reactions were performed in duplicates with a final volume of 20 μl, using 12.5 ng of cDNA, 1× *SYBR Green PCR Master Mix *(*Applied Biosystems*) and 200 or 400 nM of each primer. We used *ABI Prism 7700 Sequence Detection System *(*Applied Biosystems*) with standard temperature protocol. Primers were designed with Primer Express software V.2.0 (*Applied Biosystems*; primers sequence in Additional file 1) and the amplification efficiency (E) of each primer was calculated according to the equation E = 10^(-1/slope)^. The expression data of *TCOF1 *transcripts was determined by relative quantification in comparison to a pool of RNA from 10 controls. Four endogenous controls genes (*GAPDH*, *BCRP*, *HPRT1*, and *HMBS*), were used and their stability was verified through *geNorm VBA *applet designed for *Microsoft Excel*. This tool calculates the most stable reference genes from a set of tested candidate reference genes in a given sample panel, and calculates the gene expression normalization factor (NF) for each target sample based on the geometric mean of a user-defined number of housekeeping genes [22]. We calculated the NF for each sample based on the four endogenous controls and the expression data was calculated according to reference [23].

## Results

### Molecular characterization of the TCS patients

Pathogenic mutations were identified in 13 of the novel 15 screened individuals: five had already been reported as pathogenic [9,21,24] and eight are herein described for the first time. These new mutations were considered pathogenic because they are a nonsense mutation (c.1609C>T), disrupt splicing (c.639+1G>A) or alter the reading frame (c.3853delC, c.1298delC, c.218_222insAACC, c.4344dupA, c.431delC). Mutation c.4375_4377delAAG (TCS18) causes an in phase deletion and excludes a Lysine residue from the protein C-terminal nucleolar localization signal [25]. In Table 1, mutation and tissue availability of each patient are depicted.

### RT-PCR in leucocytes

**Figure 1:**
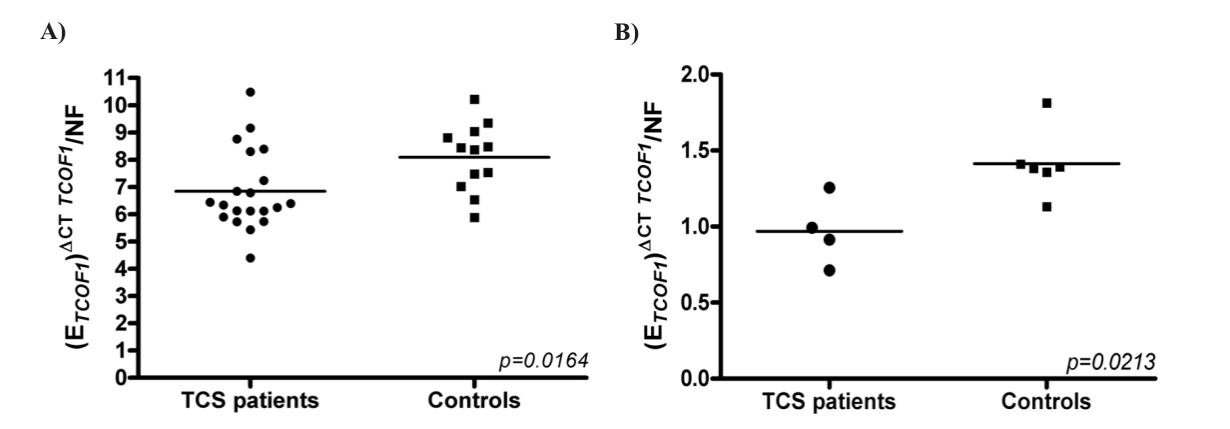
**Normalized *TCOF1 *expression levels in TCS patients (circles) and controls (squares)**. **A) ***TCOF1 *expression levels in leucocytes samples from patients (mean ± SEM = 6.846 ± 0.3278) and controls (mean ± SEM = 8.093 ± 0.3587). **B) ***TCOF1 *expression levels in mesenchymal cell samples from patients (mean ± SEM = 0.9687 ± 0.1122) and controls (mean ± SEM = 1.414 ± 0.09010). Mean expression value is represented by horizontal lines.

We first verified the range of *TCOF1 *transcript levels in leucocytes from 20 patients (Table 1) and 12 controls by real-time PCR, using as reference a pool of RNA from 10 controls. We observed a wide variation in *TCOF1 *expression in leucocytes from normal individuals (Figure 1; SD = 1.24). This wide variation of gene expression was also observed among TCS patients (Table 1; SD = 1.46), and was not correlated to age (*p = 0.39*; Two-tailed Pearson correlation regression).

Comparing patients (mean ± SEM = 6.846 ± 0.3278; N = 20) and controls (mean ± SEM = 8.093 ± 0.3587; N = 12), we observed that *TCOF1 *expression levels were significantly lower in patients than in controls (*p = 0.0164*; Two-tailed unpaired t test with Welch's correction; Figure 1), and the variances were not significantly different between these two groups (*p = 0.5828*, F test). TCS patients had approximately 18% less *TCOF1 *transcript levels than normal individuals. Excluding the four patients without an identified pathogenic mutation, we obtained similar results (~18.5%, *p = 0.0039*).

### RT-PCR in mesenchymal cells

We also examined whether *TCOF1 *expression levels were decreased in mesenchymal cells. Flow cytometry results showed that most of the cells were positive for mesenchymal cell markers (>95%) and negative for endothelial and hematopoietic markers (see Additional files 2 and 3) in primary cell cultures from four patients and four controls.

**Figure 2:**
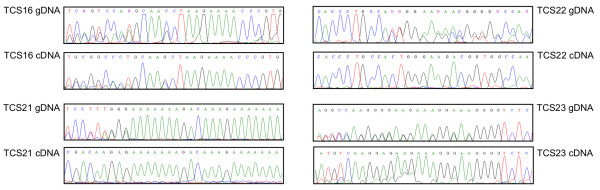
**Genomic DNA (gDNA) and complementary DNA (cDNA) sequencing of mesenchymal stem cells samples from four TCS patients**. The pathogenic mutation of TCS patients 16, 21, 22, and 23 are c.218_222insAACC (exon 3), c.4344dupA (exon 24), c.431delC (exon 5), and c.4218dupG (exon 23), respectively. gDNA was sequenced with intronic primers and cDNA with exonic primers. Observing all gDNA samples, we can assume that all analyzed individuals are heterozygous for the pathogenic mutation. Analyzing cDNA samples, we could detect the mutant allele expression in TCS 16 and 23; TCS 21 and 22 express only the wild-type allele. Note that even when the mutant allele is expressed (patients TCS16 and TCS23), the peak heights are lower.

We also observed a great variance in *TCOF1 *expression among mesenchymal cells from TCS patients (n = 4) and controls (n = 6), and the variances in these samples were also not significantly different between the groups (*p = 0.90*, F test). Although the number of tested individuals was smaller, we also detected a significantly lower expression of *TCOF1 *in TCS patients (~31%; Figure 2; patients mean ± SEM = 0.9687 ± 0.1122; controls mean ± SEM = 1.414 ± 0.09010; *p = 0.0213*; Two-tailed unpaired t test with Welch's correction). For this analysis, we also used as reference a pool of RNAs.

### Mutant allele detection

In order to confirm the existence of NMD mechanism, we evaluated the expression of mutant alleles. We compared peak heights obtained through sequencing analysis of the nucleotides flanking the pathogenic mutation of the gDNA *versus *cDNA (Figure 2). We observed the expression of the mutant alleles in two patients (TCS16 and 23), but in patients TCS21 and 22 we could only detect the wild-type allele (Figure 2).

## Discussion

We present here the first quantification of *TCOF1 *transcripts in leucocytes and mesenchymal cells from TCS patients and controls. We observed a wide range of expression levels, with a variance that was very similar for individuals with or without a pathogenic mutation in the *TCOF1 *gene, and also independently from the tissue where the mRNA was obtained. The expression levels in leucocytes did not show dependence on age in TCS patients.

We verified that the *TCOF1 *transcript levels were significantly reduced in patients as compared to controls in all the cell types studied. Our results demonstrated that the dosage compensation mechanism, proposed elsewhere [5], does not occur in adult leucocytes or mesenchymal cells, at least at the mRNA level. However, we cannot rule out the existence of post-translational regulatory mechanisms that assure the same amounts of treacle. Alternatively, the methods adopted for treacle quantification in the previous report were not able to detect a slight reduction in protein levels [5].

Our findings are in accordance with the current hypothesis of haploinsufficiency for TCS, which predicts that the allele bearing a premature stop codon is degraded by NMD, and consequently patients have less gene product than normal individuals. In addition, the reduction of *TCOF1 *expression (18-31%) in adult cells of TCS patients as compared to controls is in accordance to the observed 5-25% reduction of transcript levels caused by NMD in the presence of premature stop codon mutations in mRNA molecules [26].

In order to confirm if NMD mechanism was associated with the presence of null mutations in *TCOF1*, we evaluated if the mutant allele had reduced expression in cell culture from tissue samples of TCS patients. As we observed complete absence of mutant alleles in individuals TCS21 and 22, but partial expression in TCS23 (Figure 2), we considered that NMD was present, but with variable efficiency. Interestingly, TCS16 seemed to express both alleles in similar proportion. Although it had been described that NMD efficiency can vary according to the position of the premature stop codon [26], in these samples we did not observe any correlation between the location of the mutations at N or C-terminal and absence/reduction of mutant allele expression (Figure 2). It is possible that, in addition to NMD, other mechanisms of transcriptional regulation could be resulting in this observed differential allelic expression, such as epigenetic, environmental or stochastic events [27,28].

## Conclusions

We demonstrated that adult leucocytes and mesenchymal cells from TCS patients present significantly reduced levels of *TCOF1*. In addition, we showed that the mutant allele is much less abundant than the wild type and it might be in accordance with the hypothesis of mutant transcript degradation through NMD. Deficiency of *TCOF1 *in these cells opens a perspective to study the function of this gene in adulthood, particularly in adult stem cells. It will be important to verify if *TCOF1 *levels interfere in the renewal capacity of stem cells during bone regeneration process, as successful reconstruction of facial defects in TCS patients represents a challenge to plastic surgery.

## Abbreviations

TCS: Treacher Collins syndrome; NMD: nonsense mediated mRNA decay; PTC: premature termination codon; NF: normalization factor.

## Competing interests

The authors declare that they have no competing interests.

## Authors' contributions

CM carried out the leucocytes samples analysis, part of mutational screening and drafted the manuscript. CCO established and analyzed mesenchymal cell lines, carried out part of mutational screening. AS carried out part of mutational screening and patients recruitment. RM and AAC provided analytic and data assistance. TF and NA clinically analyzed TCS patients. MRPB provided analytical support and supervised the project. All authors have read and approved this final manuscript.

## Pre-publication history

The pre-publication history for this paper can be accessed here:

http://www.biomedcentral.com/1471-2350/10/136/prepub

## Supplementary Material

Additional file 1 Primer sequences used in this report. Click here for file

Additional file 2 Percentage of positive cells for mesenchymal cell markers. Click here for file

Additional file 3 **Flow cytometry analysis of mesenchymal cells from patients TCS16, 21, 22, and 23**. Values represent the mean percentage of all assessed cells positively stained by the indicated antigens (CD31, CD73 (SH4), (SH2) CD105) and analyzed by flow cytometry. Graphs show relative number of cells (events) versus fluorescence intensity. Unmarked cells (control) were used as negative controls in both non-conjugated and conjugated antibodies. Solid histograms (black) show marker expression; open histograms (grey) show no marker expression. Horizontal lines represent the range of positive cells interval. CD means cluster of differentiation. Click here for file
