# Supplementary material for: Reduced transcription of TCOF1 in adult cells of Treacher Collins syndrome patients
Source: BMC Med Genet. 2009 Dec 14;10:136. doi: 10.1186/1471-2350-10-136 (PMC2801500; doi:10.1186/1471-2350-10-136)
Supplement: Additional file 1 — Primer sequences used in this report. [file 1471-2350-10-136-S1.DOC]

**Additional table 1: Primer sequences used in this report (5’-3’)**

| **Real-Time PCR** | | |
| --- | --- | --- |
| **Gene** | ***Forward*** | ***Reverse*** |
| *TCOF1* | GCCCCTGGAAAAGTTGTCACT | GGTTTCTCACTGGTGGCTTCAC |
| *HMBS* | GGCAATGCGGCTGCAA | GGGTACCCACGCGAATCAC |
| *GAPDH* | ACCCACTCCTCCACCTTTGA | CTGTTGCTGTAGCCAAATTCGT |
| *HPRT1* | GAACGTCTTGCTCGAGATGTGA | TCCAGCAGGTCAGCAAAGAAT |
| *BCRP* | CCTTCGACGTCAATAACAAGGAT | CCTGCGATGGCGTTCAC |
| **cDNA sequencing** | | |
| **Covered exons** | ***Forward*** | ***Reverse*** |
| *TCOF1_3 to 5* | AACAAACCTCAGAGCTTGGTCG | TCCACGTCTGTCTCATCACTGG |
| *TCOF1_23 to 24* | GAGAGACAAAGCAAGTGGTG | ctgggacggtgactcagaat |
