# Supplementary material for: Reduced transcription of TCOF1 in adult cells of Treacher Collins syndrome patients
Source: BMC Med Genet. 2009 Dec 14;10:136. doi: 10.1186/1471-2350-10-136 (PMC2801500; doi:10.1186/1471-2350-10-136)
Supplement: Additional file 2 — Percentage of positive cells for mesenchymal cell markers. [file 1471-2350-10-136-S2.DOC]

**Additional table 2: Percentage of positive cells for mesenchymal cell markers (CD29, CD44, CD73(SH4), CD105(SH2)/SH3, HLA-ABC)**

|  | **CD29** | **CD44** | **CD73(SH4)** | **CD105(SH2)/SH3** | **HLA-ABC** |
| --- | --- | --- | --- | --- | --- |
| **TCS16** | 99,62% | 98,26% | 99,63% | 99,14% | 98,72% |
| **TCS21** | 98,38% | 72,40% | 99,08% | 98,34% | 97,20% |
| **TCS22** | 97,62% | 97,34% | 98,34% | 95,26% | 96,80% |
| **TCS23** | 99,68 | 99,74% | 97,68% | 97,80% | 99,52% |
| **Control 1** | 98,78% | 97,30% | 97,94% | 97,94% | 95,60% |
| **Control 2** | 90,68% | 84,56% | 82,88% | 84,32% | 71,48% |
| **Control 3** | 98,98% | 98,08% | 97,34% | 98,68% | 97,86% |
| **Control 4** | 81,10% | 62,50% | 88,88% | 82,80%/84,67% | 86,67% |
