# Supplementary figures and images for: Reduced transcription of TCOF1 in adult cells of Treacher Collins syndrome patients
Source: BMC Med Genet. 2009 Dec 14;10:136. doi: 10.1186/1471-2350-10-136 (PMC2801500; doi:10.1186/1471-2350-10-136)

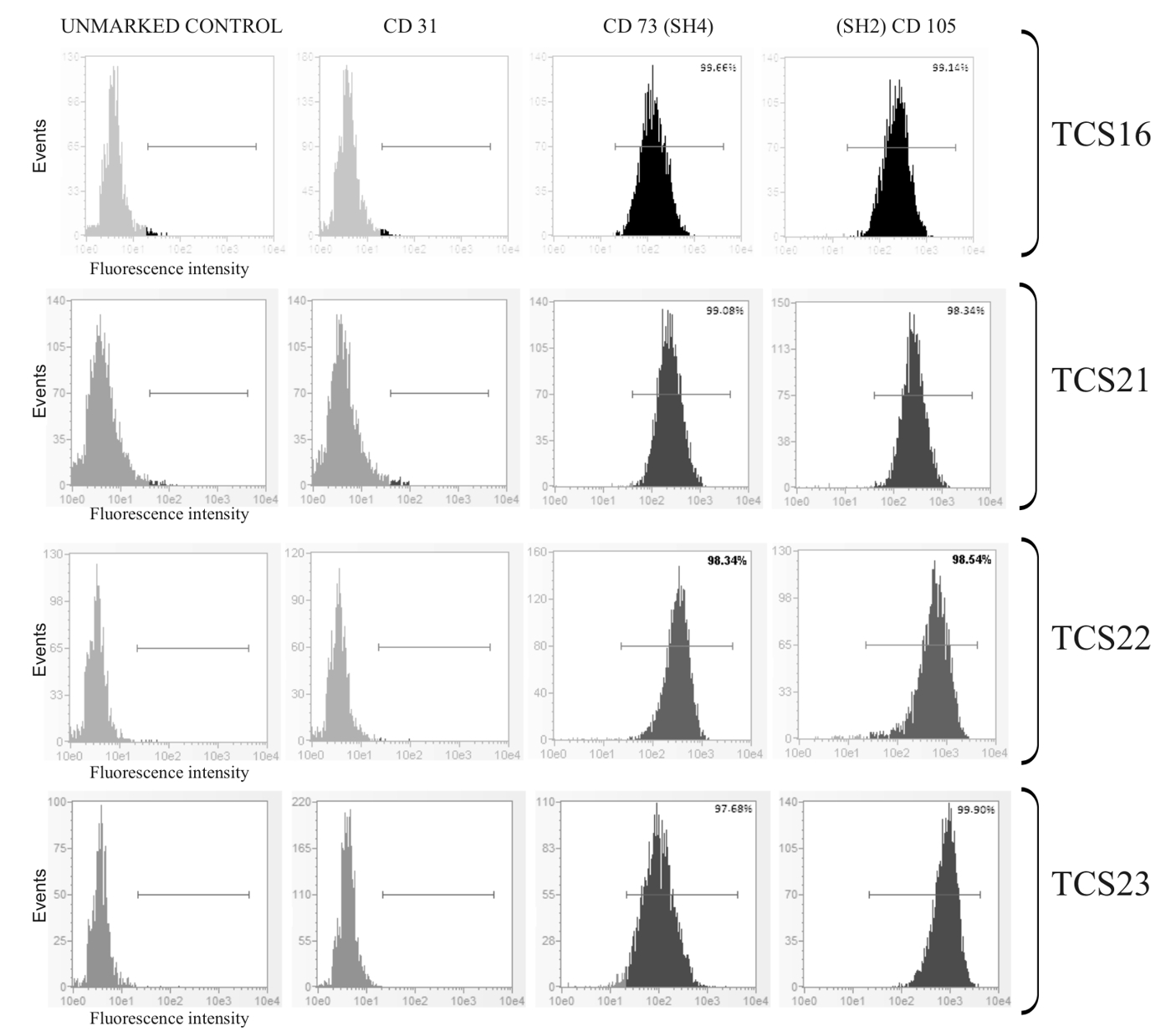

Supplement: Additional file 3 — Flow cytometry analysis of mesenchymal cells from patients TCS16, 21, 22, and 23. Values represent the mean percentage of all assessed cells positively stained by the indicated antigens (CD31, CD73 (SH4), (SH2) CD105) and analyzed by flow cytometry. Graphs show relative number of cells (events) versus fluorescence intensity. Unmarked cells (control) were used as negative controls in both non-conjugated and conjugated antibodies. Solid histograms (black) show marker expression; open histograms (grey) show no marker expression. Horizontal lines represent the range of positive cells interval. CD means cluster of differentiation. [file 1471-2350-10-136-S3.JPEG]
